# Causal effects of metabolites on malignant neoplasm of bone and articular cartilage: a mendelian randomization study

**DOI:** 10.3389/fgene.2025.1366743

**Published:** 2025-03-03

**Authors:** Yongwei Du, Xiqiu Xiao, Fuping Liu, Wenqing Zhu, Jianwen Mo, Zhen Liu

**Affiliations:** ^1^ Department of Orthopedics, First Affiliated Hospital of Gannan Medical University, Ganzhou, China; ^2^ Department of Orthopedics, 8th People Hospital of Nankang, Ganzhou, China; ^3^ Department of Emergency, Shangyou Hospital of Traditional Chinese Medicine, Ganzhou, China; ^4^ Department of Rehabilitation, First Affiliated Hospital of Gannan Medical University, Ganzhou, China

**Keywords:** mendelian randomization, causality, metabolites, neoplasm, bone, articular, cartilage

## Abstract

**Objective:**

Previous research has demonstrated that metabolites play a significant role in modulating disease phenotypes; nevertheless, the causal association between metabolites and malignant malignancies of bones and joint cartilage (MNBAC)has not been fully elucidated.

**Methods:**

This study used two-sample Mendelian randomization (MR) to explore the causal correlation between 1,400 metabolites and MNBAC. Data from recent genome-wide association studies (GWAS) involving 8,299 individuals were summarized. The GWAS summary data for metabolites were acquired from the IEU Open GWAS database, while those for MNBAC were contributed by the Finnish Consortium. We employed eight distinct MR methodologies: simple mode, maximum likelihood estimator, MR robust adjusted profile score, MR-Egger, weighted mode, weighted median, MR-PRESSO and inverse variance weighted to scrutinize the causal association between metabolites engendered by each gene and MNBAC. Consequently, we evaluated outliers, horizontal pleiotropy, heterogeneity, the impact of single nucleotide polymorphisms (SNPs), and adherence to the normal distribution assumption in the MR analysis.

**Results:**

Our findings suggested a plausible causative relationship between N-Formylmethionine (FMet) levels, lignoceroylcarnitine (C24) levels, and MNBAC. We observed a nearly significant causal association between FMet levels and MNBAC within the cohort of 1,400 metabolites (*P* = 0.024, odds ratio (OR) = 3.22; 95% CI [1.16–8.92]). Moreover, we ascertained a significant causal link between levels of C24 and MNBAC (*P* = 0.0009; OR = 0.420; 95%CI [0.25–0.70]). These results indicate a potential causative relationship between FMet, C24 level and MNBAC.

**Conclusion:**

The occurrence of MNBAC may be causally related to metabolites. This might unveil new possibilities for investigating early detection and treatment of MNBAC.

## 1 Introduction

Malignant malignancies of bones and joint cartilage (MNBAC) is a rare but severe type of tumor. The phrase “bone tumors” refers to all cancers, including primary, secondary, and metastatic tumors, originating from skeletal or other bone tissue components (Yang et al., 2023). Primary MNBAC include osteosarcoma, chondrosarcoma,malignant, lymphoma,osteofibrosarcoma, myeloma,Ewing’ssarcoma,and chordoma (Choi and Ro, 2021). MNBAC predominantly occurs in the mobile segments of the long bones, referred to as metaphysis, encompassing the proximal tibia, proximal humerus, and distal femur (Chou et al., 2014). The major clinical symptoms of MNBAC are pain, swelling, and functional impairment (Xia et al., 2018). Osteosarcoma is the most common primary MNBAC, accounting for approximately 1% of all malignancies in the United States (Suehara et al., 2019). Osteosarcoma frequently exhibits aggressive growth and metastasizes to adjacent tissues and other locations. Ewing sarcoma (ES), the second most frequent bone tumor in teenagers, flourishes in a mechanically active microenvironment (Marturano-Kruik et al., 2018), It typically occurs in children and adolescents and originates in the bone marrow or soft tissues. Conventional methods for treating bone tumors include surgical resection, radiotherapy, and chemotherapy (Beane et al., 2017). Reconstruction of the affected area post-resection is a crucial phase that significantly impacts the overall outcome and patient wellbeing (Hu et al., 2023a; Hu et al., 2023b; Hu et al., 2022). Radiotherapy may be used to reduce tumor size preoperatively, prevent recurrence after surgery, and control metastases (Jones et al., 2018). Chemotherapy is often combined with surgery and radiation therapy to eliminate potential micrometastatic lesions (Wang et al., 2019). Although malignant bone tumors are relatively rare, they pose a significant threat to the patient’s life and physical function. Therefore, exploring new targets for screening, prevention, and treatment of MNBAC is essential.

Metabolites are tiny compounds that act as intermediates and products of metabolic reactions. Multiple factors affect the levels of these metabolites, including genetics, dietary patterns, lifestyle choices, gut microbiota composition and pathological conditions (Noronha et al., 2019). Metabolites could influence the risk of maladies and be the focus of therapeutic intervention (Noronha et al., 2019). A better understanding of the causative function of metabolites in disease etiology can lead to more controllable therapeutic targets. Common genetic metabolites serve as discriminating agents in the pathogenesis of various complicated illnesses. These metabolites interact with environmental variables such as lifestyle choices, potentially influencing an individual’s susceptibility to specific disease phenotypes (Li et al., 2019). To date, GWAS has identified several metabolite-related loci in human urine and blood specimens (Cai et al., 2021). Moreover, these loci correlated with the progression and prognosis of respiratory disorders (Chang et al., 2023), gastrointestinal maladies (Kim et al., 2019), cardiovascular conditions (Mihuta et al., 2023), endocrine dysregulation (Tan et al., 2023), as well as tumor diseases (Gubser and Kallies, 2020). However, limited studies have investigated the association between 1,400 metabolites and MNBAC.

Using GWAS, we can scrutinize genetic variations within extensive populations and juxtapose them with diverse metabolite concentrations, disease ramifications, and other pertinent attributes to elucidate the involvement of metabolites in disease consequences (Tang et al., 2019). Numerous metabolite levels have shown high heritability, providing the opportunity to perform Mendelian randomization (MR) (Civelek and Lusis, 2014). MR is an instrumental variable analysis approach utilizing genetic variations as tools to evaluate causal connections between potentially modifiable exposures, such as single nucleotide polymorphisms (SNPs), and clinically significant outcomes; it has been extensively employed to investigate causal inference in epidemiological studies (Liu et al., 2023a; Liu et al., 2023b; Xiang et al., 2021).

This study explored the causal relationship between 1,400 metabolites and MNBAC employing MR analysis coupled with metabolomics using GWAS data of MNBAC as the outcome file and GWAS data of 1,400 metabolites as the exposure file. Furthermore, this study identified relevant metabolites, providing novel insights into early detection and therapeutic strategies for MNBAC.

## 2 Methods

### 2.1 The flowchart and assumption of MR

**FIGURE 1:**
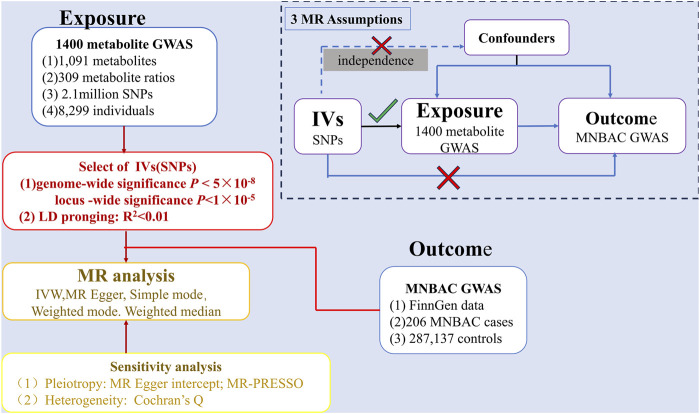
MR analyses process and major assumptions.

The causal links between 1,400 metabolites and MNBAC were examined using a two-sample MR analysis. Summary-level GWASs data were used for the metabolites and MNBAC. The flowchart of this study is displayed in Figure 1. Furthermore, to ensure the accuracy of the findings, the MR analysis must adhere to three fundamental hypotheses: (1) The instrumental variables (IVs) employed exhibited a robust association with metabolites. (2) The selected IVs and confounding factors that influenced both the metabolites and MNBAC were mutually independent. (3) The absence of horizontal pleiotropy was ensured: IVs solely influenced MNBAC through metabolites (Davey Smith and Hemani, 2014) (Figure 1). Moreover, the results obtained were reported following the MR-STROBE protocol (Choi et al., 2022).

### 2.2 Exposure sources of 1,400 metabolites

Metabolic data were derived from the extensive GWAS analysis conducted by Chen et al. in the esteemed journal “Nature Genetics” (Chen et al., 2023). This investigation amalgamated 309 metabolite ratios and 1,091 individual metabolites from a cohort of 8,299 participants within the esteemed Canadian Longitudinal Study of Aging (CLSA). The CLSA cohort comprised nearly 2.1 million SNPs and 452 blood metabolites. Comprehensive GWAS summary statistics are accessible for direct retrieval from the European GWAS (GWAS ID: met-a) under the accession number GCST90199621-902010209, encompassing data for 1,400 metabolites.

### 2.3 Outcome sources of MNBAC

The GWAS summary data for MNBAC were obtained from the FinnGen studies, which are available through their website (https://r9.finngen.fi/) and included individuals of European ancestry, both men and women. SAIGE (https://github.com/weizhouUMICH/SAIGE) was utilized to conduct the GWAS analysis, incorporating 20175454 variable SNPs across a cohort of 377,277 participants. After adjustments for factors such as age, gender, high genotypic individual deletions (>5%), excessive heterozygosity (4SD), and non-Finnish lineage, a subset of 206 MNBAC cases and 287,137 controls were selected for scrutiny. MNBAC was defined using the ICD-10 code M13. Further information on the data can be found on the FinnGen website.

### 2.4 Statistical analysis

Statistical analysis was executed utilizing R software (version 4.3.1). The “TwoSampleMR” software was employed to perform MR analysis of the causal relationship between metabolites and MNBAC. *P* < 0.05 ordinarily signifies the statistically significance of the findings, thus indicating that such a correlation may be regarded as evidence of causality (Xiang et al., 2021).

#### 2.4.1 IVs selection

Meticulous selection of the approved IVs was imperative for enhancing the robustness of MR analysis. Initially, we pursued stringent criteria characterized by formidable values of 1 × 10^−5^ and 5 × 10^−8^. The SNPs used in the MR test adhered to the principles of Mendelian inheritance: parental alleles were randomly allocated to offspring, impervious to acquired traits. Therefore, these alleles exhibited a high degree of independence and were potentially unrelated to confounding factors. The universal standards for SNP screening encompassed two thresholds: *P* < 1 × 10^−5^ and *P* < 5 × 10^−8^, signifying their statistically significant inclusion in the research. Lastly, we used Steiger filtration to eliminate any IVs that may lead to causal inversion.

#### 2.4.2 Statistical analyses for MR

We examined the two cohorts using 1,400 metabolites as the exposure and MNBAC as the outcome in this study. MR analyses were executed using the “Two Sample MR” software package, with IVW analysis employed to synthesize the effects of multiple loci and evaluate numerous SNPs (Yan et al., 2023). Without horizontal pleiotropy, the IVW test was used as the principal method for assessing causal effects to obtain unbiased estimates (Du et al., 2023). The presence or absence of heterogeneity determined the existence of fixed or random effects. The effect estimates were presented as odds ratios (ORs) and 95% confidence intervals (CI).

In addition to MR analysis, the maximum likelihood estimator (MLE), MR robust adjusted profile score (MR-RAPS), MR-Egger test (Bowden et al., 2015) and the weighted median (WM) approach (Bowden et al., 2016) were employed. WM data were utilized to determine substantial causation. The absence of horizontal pleiotropy was established if *P* > 0.05. The basic model and MR-PRESSO analyses were used as part of the sensitivity analyses (Liu K. et al., 2022). The F statistic was calculated using aggregated data levels to ascertain IV exposure correlations. If F > 10, the correlations were considered sufficiently robust to mitigate the weak IVs bias. Within the IVW framework, the Cochrane Q statistic was utilized to evaluate heterogeneity among SNP estimates. Additionally,we validated the robustness of the data using the simple mode and the leave-one-out method (Liu K. et al., 2022).

## 3 Result

### 3.1 The study design of MR

The causal links between MNBAC and 1,400 metabolites were unveiled through a two-sample MR analysis. The categorization of metabolites and MNBAC conformed to the aggregated data acquired from the GWASs. Figure 1 depicts the flowchart outlining the MR investigation involving the metabolites and MNBAC.

### 3.2 Selection of IVs related to MNBAC

We meticulously selected IVs linked to MNBAC from a pool of 2.1 million SNPs associated with 1,400 metabolites. Subsequent to a quality control procedure integrating the Linkage Disequilibrium (LD) effect and retrogression method, we utilized a *P* < 1 × 10^−5^ for the calculations, resulting in the identification of 30,276 SNPs, 2295 SNP-metabolites, and IVs for MNBAC (threshold 1 × 10^−5^). Each SNP demonstrated adequate validity (F-values ranging from 19.51 to 2,298.39, all F > 10) (Table 1). The most significant information of the IVs is presented in Supplementary Table S1 (*P* < 1 × 10^−5^). Additionally, to establish the robustness of the results, we adopted a more stringent threshold of 5 × 10^−8^ for the analysis, which identified 2295 SNP metabolites and IVs for MNBAC (F-values ranging between 29.71 and 2,298.39, all F > 10). Significant data regarding IVs were provided within the specifics. Supplementary Table S2 outlines the primary information of the IVs (*P* < 5 × 10^−8^).

**TABLE 1:** Causal results of MR analysis between metabolites and MNBAC with threshold of *P* < 1 ⅹ 10^–5^.

Exposure	method	nsnp	pval	Or (95%CI)
N-formylmethionine levels	MR Egger	20	13.46 × 10^−2^	3.19 (0.74, 13.60)
N-formylmethionine levels	Weighted median	20	0.14 × 10^−2^	3.79 (1.67, 8.59)
N-formylmethionine levels	Inverse variance weighted	20	2.36 × 10^−2^	2.04 (1.10, 3.78)
N-formylmethionine levels	Simple mode	20	63.71 × 10^−2^	0.66 (0.11, 3.64)
N-formylmethionine levels	Weighted mode	20	1.60 × 10^−2^	3.65 (1.39, 9.53)
Isoursodeoxycholate levels	MR Egger	17	1.09 × 10^−2^	0.18 (0.05, 0.57)
Isoursodeoxycholate levels	Weighted median	17	6.90 × 10^−2^	0.31 (0.13, 0.72)
Isoursodeoxycholate levels	Inverse variance weighted	17	4.43 × 10^−2^	0.49 (0.24, 0.98)
Isoursodeoxycholate levels	Simple mode	17	15.02 × 10^−2^	0.27 (0.04, 1.47)
Isoursodeoxycholate levels	Weighted mode	17	3.67 × 10^−2^	0.22 (0.06, 0.81)
Methionine sulfone levels	MR Egger	29	4.69 × 10^−2^	1.97 (1.04, 3.73)
Methionine sulfone levels	Weighted median	29	3.24 × 10^−2^	1.74 (1.04, 2.92)
Methionine sulfone levels	Inverse variance weighted	29	4.80 × 10^−2^	1.42 (1.00, 2.03)
Methionine sulfone levels	Simple mode	29	30.17 × 10^−2^	1.75 (0.62, 5.01)
Methionine sulfone levels	Weighted mode	29	3.92 × 10^−2^	1.78 (1.05, 3.02)
Methyl glucopyranoside (alpha + beta) levels	MR Egger	22	5.72 × 10^−2^	1.41 (1.01, 1.96)
Methyl glucopyranoside (alpha + beta) levels	Weighted median	22	4.41 × 10^−2^	1.45 (1.01, 2.09)
Methyl glucopyranoside (alpha + beta) levels	Inverse variance weighted	22	0.30 × 10^−2^	1.49 (1.14, 1.93)
Methyl glucopyranoside (alpha + beta) levels	Simple mode	22	8.12 × 10^−2^	2.15 (0.94, 4.89)
Methyl glucopyranoside (alpha + beta) levels	Weighted mode	22	2.71 × 10^−2^	1.40 (1.06, 1.86)
Lignoceroylcarnitine (C24) levels	MR Egger	21	0.66 × 10^−2^	0.27 (0.12, 0.63)
Lignoceroylcarnitine (C24) levels	Weighted median	21	0.77 × 10^−2^	0.48 (0.28, 0.82)
Lignoceroylcarnitine (C24) levels	Inverse variance weighted	21	0.06 × 10^−2^	0.50 (0.34, 0.74)
Lignoceroylcarnitine (C24) levels	Simple mode	21	93.49 × 10^−2^	0.96 (0.37, 2.47)
Lignoceroylcarnitine (C24) levels	Weighted mode	21	3.26 × 10^−2^	0.52 (0.29, 0.90)

### 3.3 MR analyses results (*P* < 1 × 10^−5^)

**FIGURE 2:**
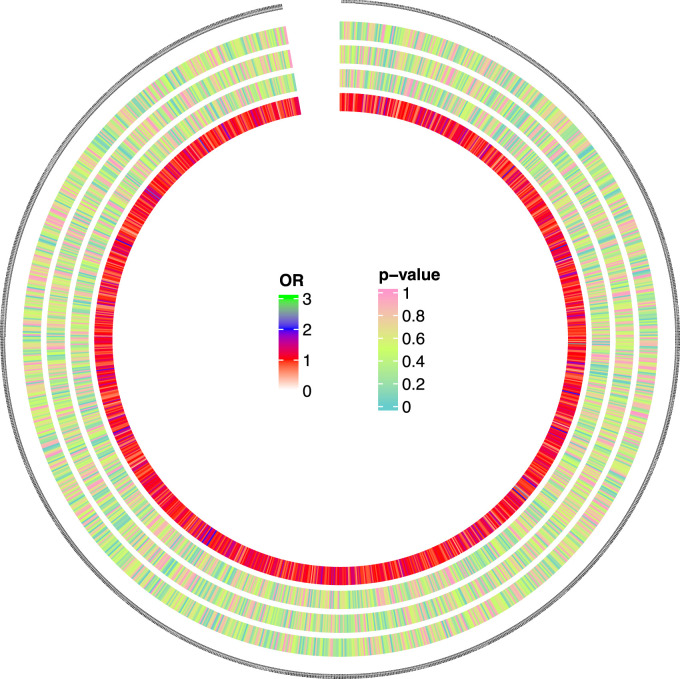
Causal analysis 562 results of 1,400 metabolites and MNBAC (locus-wide significance, *P* < 1 × 10^−5^). The color corresponding to the *P* value is based on the RGB color (*P* = 0, #66CCCC; *P* = 0.5, #CCFF66; *P* = 1, #FF99CC). The color corresponding to the OR value is based on the RGB color (OR = 0, white; OR = 1, red; OR = 2, blue; OR = 3, green).

We evaluated the influence of 1,400 metabolites on bone tumor risk at a threshold of 1 × 10^−5^, and found suggestive evidence of causality (*P* < 0.05) for five metabolites. These included N-formylmethionine (FMet) levels (*P* = 0.001; OR = 3.789; 95%CI [1.670–8.593]),isoursodeoxycholate levels (*P* = 0.010; OR = 0.183; 95% CI [0.058–0.576]), methionine sulfone levels (*P* = 0.032; OR = 1.749; 95%CI [1.048–2.921]), methyl glucopyranoside (alpha + beta) levels (*P* = 0.044; OR = 1.451; 95% CI [1.009–2.085]), and lignoceroylcarnitine (C24) levels (*P* = 0.006, OR = 0.268; 95% CI [0.115–0.625]). Table 1 and Figure 2 presented these findings. Notably, three of these metabolites, namely, methyl glucopyranoside (alpha + beta) levels, fMet levels, and methionine sulfone levels, were particularly associated with high-risk factors for bone and joint cancer. Additionally, isoursodeoxycholate concentrations and C24 levels, might be linked to low-risk bone tumor. These findings were validated using five different methods (Supplementary Table S3).

### 3.4 Heterogeneity analysis (*P* < 1 × 10^−5^)

**FIGURE 3:**
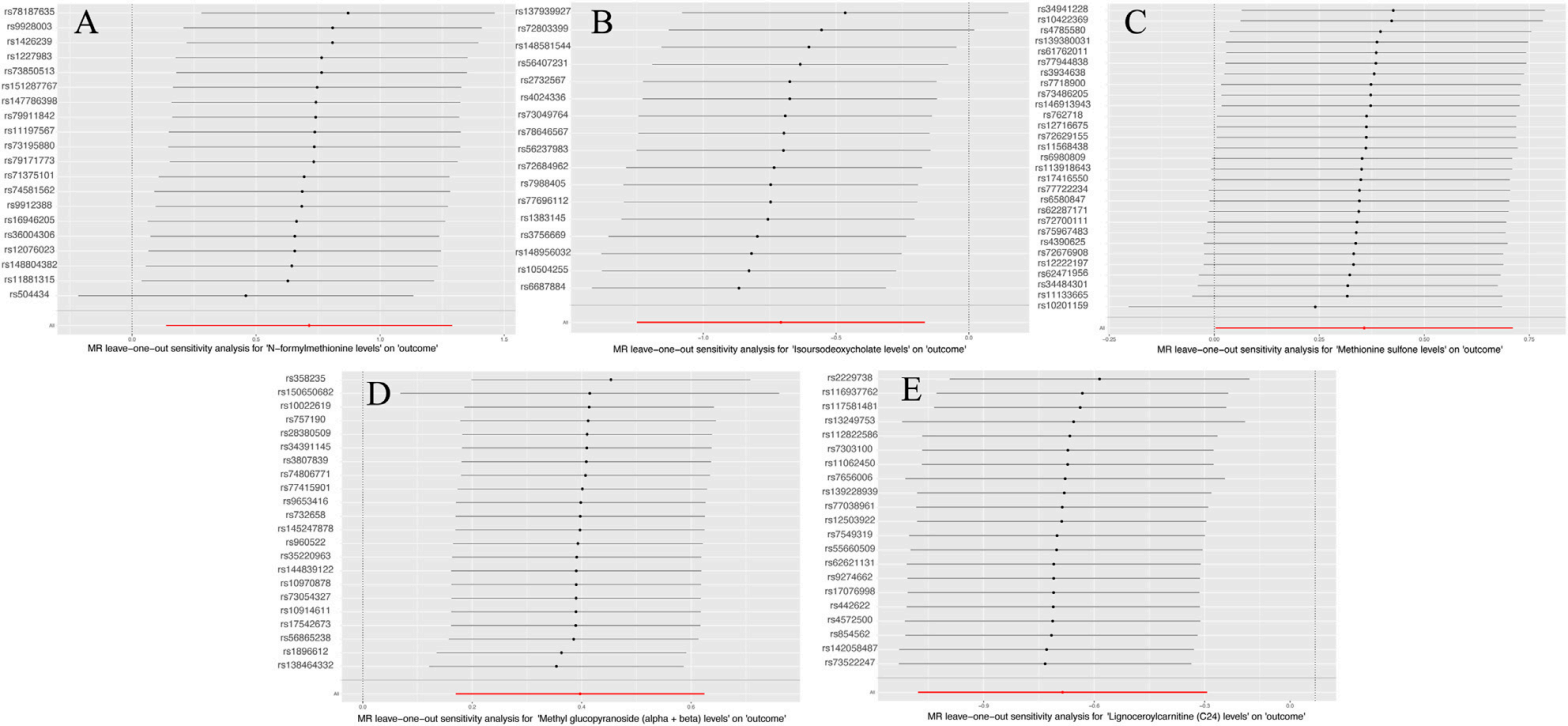
The leave-one-out results of 1,400 metabolites and MNBAC (locus-wide significance, *P* < 1 × 10^−5^). **(A)** N-formylmethionine levels **(B)**. Isoursodeoxycholate levels **(C)**. Methionine sulfone levels **(D)**. Methyl glucopyranoside (alpha + beta) levels **(E)**. Lignoceroylcarnitine (C24) levels.

Supplementary Table S5 lists the results of multiplicity and heterogeneity assessments for all metabolites. Through sensitivity analyses,we verified the effect of accurate MR results on metabolites of MNBAC. Notably, FMet levels (*P* = 0.51), Isoursodeoxycholate levels (*P* = 0.06), Methionine sulfone levels (*P* = 0.24), Methyl glucopyranoside (alpha + beta) levels (*P* = 0.58), and C24 levels showed no evidence of horizontal pleiotropy in relation to bone tumors (P = 0.11) (Table 2). Meanwhile, no heterogeneity was observed in FMet levels (MR-Egger: *P* = 0.26; IVW: P = 0.29), Isoursodeoxycholate levels (MR-Egger: *P* = 0.15; IVW: *P* = 0.05), Methionine sulfone levels (MR-Egger: *P* = 0.55; IVW: *P* = 0.53), and Methyl glucopyranoside (alpha + beta) levels (MR-Egger: *P* = 0.12; IVW: *P* = 0.13) (Table 2). Furthermore, the leave-one-out analysis showed no meaningful difference. in casual estimation of FMet levels. Methionine sulfone levels Isoursodeoxycholate levels. Methyl glucopyranoside (alpha + beta) levels and C24 levels on MNBAC (Figure 3).

**TABLE 2:** MR results of sensitivity analysis with threshold of *P* < 1 ⅹ 10^–5^.

Exposure	Method	Q	Q- pval	Method	Q	Q_pval	egger_intercept	pval	MR-PRESSO
N-formylmethionine levels	IVW	21.85	0.29	MR Egger	21.32	0.26	−0.06	0.51	0.29
Isoursodeoxycholate levels	IVW	25.92	0.05	MR Egger	20.34	0.16	0.143	0.06	0.07
Methionine sulfone levels	IVW	26.70	0.53	MR Egger	25.29	0.55	−0.05	0.24	0.62
Methyl glucopyranoside (alpha + beta) levels	IVW	28.05	0.13	MR Egger	27.63	0.11	0.02	0.58	0.23
LC (C24) levels	IVW	18.32	0.56	MR Egger	15.61	0.68	0.11	0.11	0.51

To validate the accuracy of MR Egger regression, we further validated the significant MR results using MLE, MR-PRESSO, MR-RAPS. We found no evidence of heterogeneity in FMet levels (*P* = 0.295), isoursodeoxycholate levels (*P* = 0.074), methionine sulfone levels (*P* = 0.622), methyl glucopyranoside (alpha + beta) levels (*P* = 0.238), and C24 levels (*P* = 0.519), indicating the lack of horizontal pleiotropy (Table 2). Moreover, data robustness was reinforced through sample-by-sample exclusion analysis, which demonstrated consistent IVW results for lack of heterogeneity and pleiotropy. Based on these findings, there appeared to be a suggestive causal correlation between FMet levels, isoursodeoxycholate levels, methionine sulfone levels, methyl glucopyranoside (alpha + beta) levels, and C24 levels with MNBAC.

### 3.5 Results of MR analysis (*P* < 5 × 10^-8^)

**FIGURE 4:**
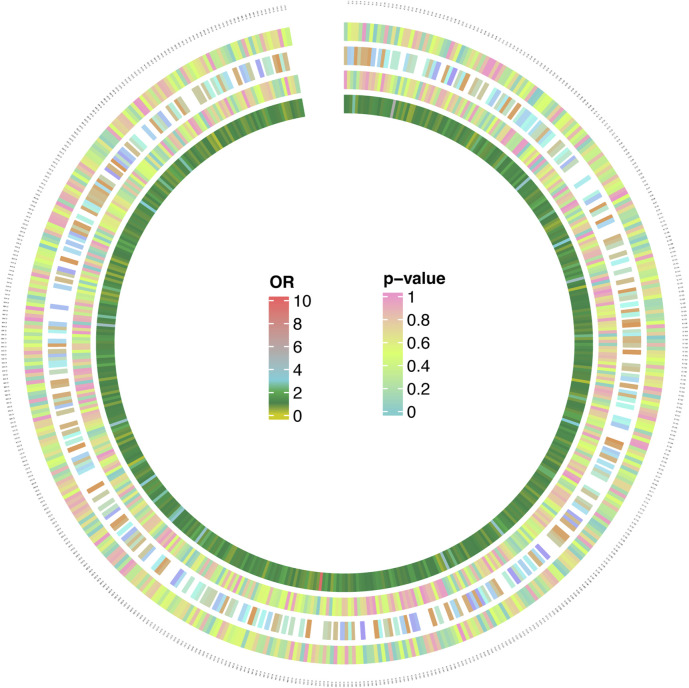
MR analysis 566 results of 1,400 metabolites and MNBAC (genome-wide statistical significance, *P* < 5 × 10^−8^). The color corresponding to the *P* value is based on the RGB color (*P* = 0, #66CCCC; *P* = 0.5, #CCFF66; *P* = 1, #FF99CC). The color corresponding to the OR value is based on the RGB color (OR = 0, #CCCC00; OR = 1, #088247; OR = 2, #11AA4D; OR = 3, #58CDD9; OR = 10, #FF6666).

Supplementary Table S4 presents the results pertaining FMet levels and MNBAC, illustrating a notable causal significance for FMet levels in MR analyses (IVW: OR = 3.12, 95%CI [1.16–8.92], *P* = 0.05; WM: OR = 3.22, 95%CI [1.16–8.92], *P* = 0.02; MR Egger: OR = 55.32, 95%CI [1.74–1750.53], *P* = 0.26). Furthermore, there was a significant causal relationship between the metabolite levels of C24 and MNBAC (IVW: OR = 0.42; 95%CI [0.25–0.70]; *P* = 0.0009; WM: OR = 0.47; 95% CI [0.26–0.85]; *P* = 0.01; MR Egger: OR = 0.42; 95%CI [0.26–0.85]; *P* = 0.01) (Table 3; Figure 4).

**TABLE 3:** Causal Results of MR analysis between metabolites and MNBAC with threshold of *P* < 5 ⅹ 10^–8^.

Exposure	Method	nsnp	pval	Or (95%CI)
N-formylmethionine levels levels	MR Egger	3	26.34 × 10^−2^	55.32 (1.74, 1750.53)
N-formylmethionine levels levels	Weighted median	3	2.40 × 10^−2^	3.22 (1.16, 8.92)
N-formylmethionine levels levels	IVW	3	5.18 × 10^−2^	3.12 (0.99, 9.87)
N-formylmethionine levels levels	Simple mode	3	17.29 × 10^−2^	5.55 (1.10, 27.95)
N-formylmethionine levels levels	Weighted mode	3	16.06 × 10^−2^	3.90 (1.14, 13.27)
Lignoceroylcarnitine (C24) levels	MR Egger	4	42.81 × 10^−2^	0.42 (0.07, 2.31)
Lignoceroylcarnitine (C24) levels	Weighted median	4	13.57 × 10^−2^	0.47 (0.26, 0.85)
Lignoceroylcarnitine (C24) levels	IVW	4	0.09 × 10^−2^	0.42 (0.25, 0.70)
Lignoceroylcarnitine (C24) levels	Simple mode	4	19.35 × 10^−2^	0.49 (0.22, 1.12)
Lignoceroylcarnitine (C24) levels	Weighted mode	4	10.33 × 10^−2^	0.47 (0.25, 0.89)

### 3.6 Heterogeneity analysis (*P* < 5 × 10^−8^)

**FIGURE 5:**
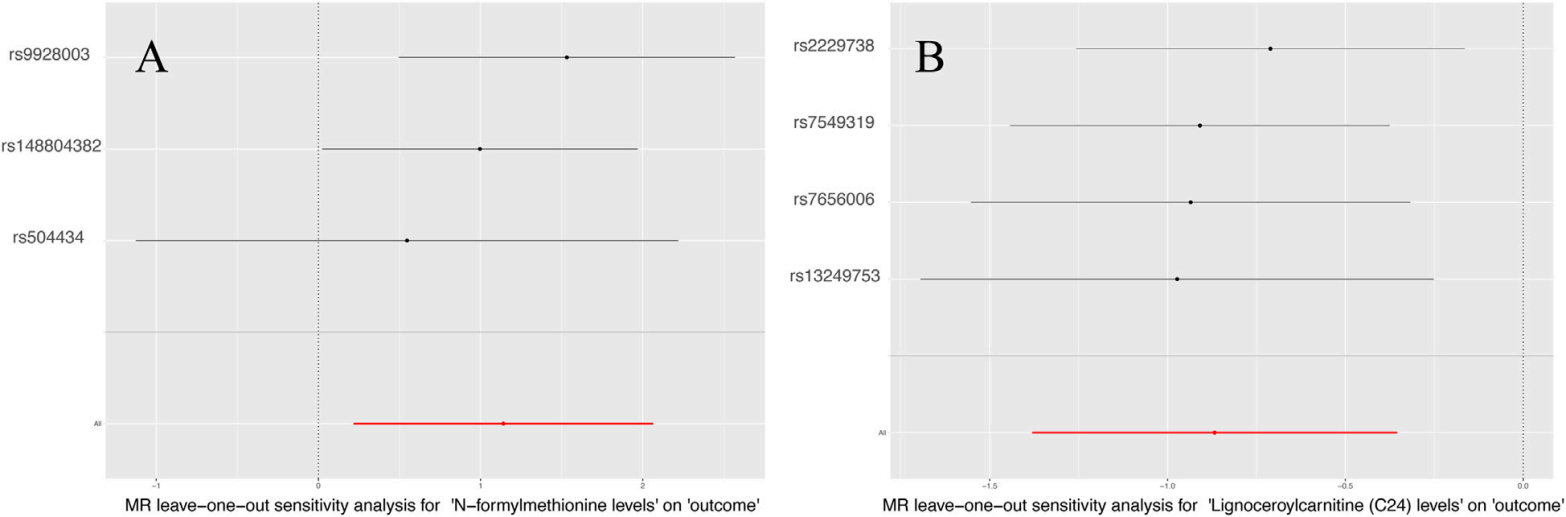
The leave-one-out results of 1,400 metabolites and MNBAC (P < 5 × 10^−8^); **(A)** N-formylmethionine levels **(B)**. Lignoceroylcarnitine (C24) levels.

Supplementary Table S6 displays the pleiotropy and heterogeneity test results for metabolisms. Heterogeneity analysis results of C24 levels (MR Egger: *P* = 0.24; IVW: *P* = 0.41) and multiplicity analysis (MR Egger: *P* = 0.34; MR-PRESSO: *P* = 0.31) verified reliability of the results. Likewise, the scrutiny of heterogeneity in FMet levels (MR Egger: *P* = 0.62; IVW: *P* = 0.21) and the multiplicity analysis (MR-PRESSO: NA; MR Egger: *P* = 0.34) verified the accuracy of the data (Table 4). Concurrently, the findings of sample-by-sample exclusion further validated the robustness of the data (Figure 4). Unfortunately, due to the overly stringent 5 × 10^−8^ threshold, only FMet levels and C24 levels were obtained with fewer instrumental variables. Notably, C24 levels exhibited a significant correlation with MNBAC, while FMet levels approached significance in terms of causal inference. In addition,the leave-one-out analysis showed some difference. in casual estimation of FMet levels and C24 levels on MNBAC (Figure 5; Supplementary Figure S2). Furthermore, due to an insufficiency of IVs, the multiplicity assessment for FMet levels was unattainable via MR-PRESSO.

**TABLE 4:** MR results of sensitivity analysis, with threshold of *P* < 5 ⅹ 10^–8^.

Exposure	Method	Q	Q_pval	Method	Q	Q_pval	Egger intercept	pval	MR-presso
N-fet levels	IVW	3.09	0.21	MR Egger	0.23	0.62	−0.46	0.33	NA
LC (C24) levels	IVW	2.82	0.41	MR Egger	2.82	0.24	−0.004	0.98	0.30

### 3.7 Further validation of the MR results

To further ascertain the causal relationship between metabolites and MNBAC, we employed additional methods to validate the results. Under the threshold of *P* < 1 × 10^−5^, the outcomes of MR-PRESSO, MR-RAPS, and MLE provided additional substantiation of the causal nexus between FMet levels, Methionine sulfone levels, Isoursodeoxycholate levels, C24 levels, and Methyl glucopyranoside (alpha + beta) levels with bone tumors (Table 5). The results from MR-RAPS and MLE confirmed the causal relationship between FMet levels and MNBAC, albeit not verified by MR-PRESSO. Meanwhile, C24 levels were further validated by MR-PRESSO, MR-RAPS, and MLE at a threshold of *P* < 5 × 10^−8^ (Table 6).

**TABLE 5:** MR Results of sensitivity analysis with threshold of *P* < 1 ⅹ 10^–5^.

Exposure	Method	Or (95%CI)	p-val
N-formylmethionine levels	MR-PRESSO	1.93 (1.43, 2.63)	4.31 × 10^−2^
N-formylmethionine levels	MR-RAPs	1.98 (1.47, 2.67)	2.19 × 10^−2^
N-formylmethionine levels	MLE	1.97 (1.47, 2.65)	2.07 × 10^−2^
Methionine sulfone levels	MR-PRESSO	1.46 (1.24, 1.72)	2.87 × 10^−2^
Methionine sulfone levels	MR-RAPs	1.47 (1.23, 1.76)	3.08 × 10^−2^
Methionine sulfone levels	MLE	1.47 (1.23, 1.75)	2.89 × 10^−2^
Isoursodeoxycholate levels	MR-PRESSO	0.51 (0.36, 0.71)	5.93 × 10^−2^
Isoursodeoxycholate levels	MR-RAPs	0.49 (0.37, 0.65)	1.08 × 10^−2^
Isoursodeoxycholate levels	MLE	0.52 (0.39, 0.68)	1.84 × 10^−2^
Lignoceroylcarnitine (C24) levels	MR-PRESSO	0.52 (0.43, 0.62)	1.03 × 10^−3^
Lignoceroylcarnitine (C24) levels	MR-RAPs	0.51 (0.42, 0.61)	2.81 × 10^−4^
Lignoceroylcarnitine (C24) levels	MLE	0.52 (0.43, 0.62)	3.16 × 10^−4^
Methyl glucopyranoside (alpha + beta) levels	MR-PRESSO	1.48 (1.30, 1.69)	6.31 × 10^−3^
Methyl glucopyranoside (alpha + beta) levels	MR-RAPs	1.49 (1.31, 1.70)	1.93 × 10^−3^
Methyl glucopyranoside (alpha + beta) levels	MLE	1.50 (1.32, 1.70)	1.33 × 10^−3^

**TABLE 6:** MR results of sensitivity analysis with threshold of *P* < 5 ⅹ 10^–8^.

Exposure	Method	Or (95%CI)	P-val
N-formylmethionine levels	MR-PRESSO	Not enough intrumental variables	NA
N-formylmethionine levels	MR-RAPs	3.19 (1.96, 5.19)	1.69 × 10^−2^
N-formylmethionine levels	MLE	3.19 (1.97, 5.19)	1.66 × 10^−2^
Lignoceroylcarnitine (C24) levels	MR-PRESSO	0.48 (0.35, 0.66)	8.26 × 10^−2^
Lignoceroylcarnitine (C24) levels	MR-RAPs	0.48 (0.37, 0.62)	3.70 × 10^−3^
Lignoceroylcarnitine (C24) levels	MLE	0.48 (0.37, 0.62)	3.70 × 10^−3^

### 3.8 Ethics statement

This summary-level data utilized in this study are de-identified public data and are accessible to download. Each GWAS in this study received ethical approval from their respective universities.

## 4 Discussion

This research conducted an MR analysis to investigate the potential causal relationship between 1,400 metabolites and MNBAC. By investigating the association from a host genetic perspective, we aimed to validate the role of these metabolites in altering susceptibility to MNBAC. Five MR methods were employed for the analysis. Although some of the results from various analytical approaches were inconsistent, these differences did not significantly influence our findings. The random effects IVW technique exhibited superior statistical power compared to the other approaches, hence it was selected as the major analytical approach in this work. While there was a potential causal relationship, and multiple corrections are too strict, they were also close to being corrected. The results of this study suggested that the two metabolites may be linked to a lower risk of MNBAC, while the three metabolites are related to a higher risk of MNBAC. Our findings open up possibilities for identifying novel biomarkers that can be utilized in future MNBAC studies. Moreover, our results indicated potential avenues for MNBAC prevention and treatment, including the targeted manipulation of specific metabolite levels. Notably,the cross-sectional aspect of this study made it difficult to find a definitive connection between metabolites and MNBAC. However, using MR analysis, we provided valuable insights into the potential causative association and highlight the significance of these metabolites in influencing susceptibility to MNBAC.

As the most prevalent type of MNBAC, osteosarcoma generates severe symptoms and poses a threat to individuals of all ages due to malignant neoplasia (Quintero Escobar et al., 2020). Several studies on osteosarcoma have examined aberrant metabolisms. The development and progression of osteosarcoma is closely related to cellular metabolites (Velayutham et al., 2023). A promising natural metabolite, stylolite, has been discovered to activate vascular endothelial growth factor receptor 2 (VEGFR2)and trigger its downstream signaling pathways. This activation promotes endothelial cell proliferation and angiogenesis while hindering the growth and invasion of osteosarcoma cells, simultaneously enhancing the sensitivity to chemotherapy drugs. Moreover, particular metabolites such as zoledronic acid induce iron-induced death in osteosarcoma cells by reducing coenzyme Q levels and stimulating heme oxygenase 1(HMOX1)expression (Ren et al., 2022). *In vitro* experiments involving osteosarcoma stem cells have revealed comparable declines in metabolites associated with the tricarboxylic acid (TCA) cycle (Zhong et al., 2019). These reductions stem from impaired mitochondrial function and are accompanied by diminished glutamine, aspartate, and glutathione levels (Ren et al., 2020; Zhong et al., 2019). Metabolite-based biomarkers for osteosarcoma exhibit potential for diagnosis and monitoring disease progression (Fan et al., 2021). These substances have been linked to developing and regulating glucose metabolism and cellular regulatory mechanisms in osteosarcoma. In conclusion, metabolites play a vital role in MNBAC research. Here, we evaluated the effects of 1,400 metabolites on MNBAC risk and identified five metabolites that showed suggestive causal relationships with MNBAC, which were validated using more than five methods. These metabolites included the FMet levels, methionine sulfone levels, methyl glucopyranoside (alpha + beta) levels, isoursodeoxycholic acid levels, and C24 levels. Among them, our finding of significant causality between C24 levels and MNBAC is novel. The FMet levels showed near-significant results. This study is the first to discover the correlation between 1,400 metabolites and MNBAC.

C24 is a long-chain fatty acid derivative that participates in the metabolism of fatty acids, particularly in the beta-oxidation process within the mitochondria (Abd-Allah et al., 2009). Abnormal fatty acid metabolism may be associated with tumor growth and survival, thus, changes in C24 levels may reflect alterations in tumor metabolic status in osteosarcoma cells, Researchers have found a link between the concentration of C24 in serum and the risk of MNBAC development, with higher concentrations associated with lower risks (Liu T. et al., 2022), Similarly, a study on the testing dose of C24 reported that it can reverse fatigue symptoms in MNBAC patients with C24 deficiency (Farahzadi et al., 2023). Our research has identified a noteworthy inverse relationship between C24 levels and MNBAC, indicating a possible function of C24 in inhibiting tumor growth. Assessing C24 levels could potentially assist in identifying patients at high risk of MNBAC and serve as a biomarker for tracking disease progression and evaluating treatment efficacy.

FMet, an amino acid that typically corresponds to the start codon, signifies the initiation of polypeptide chain synthesis. The role of FMet in protein degradation processes is also significant, particularly in the activity of peptidyl deformalize (PDF) (Silver, 2011). In osteosarcoma cells, PDF activity may be upregulated, thereby affecting protein stability and intracellular signal transduction (Lee et al., 2004; Pietzke et al., 2020). In our study, the significant positive correlation between the FMet levels and MNBAC indicated a potential role of FMet in promoting tumor growth. Similarly, in another 11,966 individuals, FMet levels may resulted in all-cause mortality and the risk of human cancer. including MNBAC. suggesting a substantial connection between FMet and the risk of MNBAC (Cai et al., 2021), In another cancer study, FMet was utilized as a drug precursor, converted into formic acid through the activity of PDF enzyme (Yu et al., 2015).

This study had several limitations. First, like other MR researches on metabolites, although our study satisfies the MR assumptions (IVs is closely related to the metabolite), there may be other mechanisms or factors in some cases that result in a correlation between IVs and the target variable, rather than a causal relationship. Second, our study’s sample sizes was modest, which may alter the dependability of our results. Given that GWAS only included European ancestry participants, our findings may not be applicable to other racial populations. Third, the multiple statistical correction employed was overly strict and conservative, potentially overlooking the metabolites that may have a causal relationship with MNBAC. While there is potential for a causal relationship, and the multiple corrections were overly strict, they approach correction. Therefore, we considered biological plausibility and did not rely solely on the results of the multiple-hypothesis testing. Finally, due to the insufficient availability of an ample number of IVs in this study, the implementation of reverse MR analysis and multivariable Mendelian randomization analyses (MVMR) was precluded. In future research, we plan to undertake GWAS investigations specifically targeting FMet levels and C24 levels to secure a robust set of IVs. This will facilitate a more thorough validation of the causal relationship between metabolites and MNBAC through the application of reverse MR analysis and MVMR.

Concludingly, this study confirmed the causal link between metabolites and MNBAC species, including and FMet levels. These metabolites have the potential to serve as new biomarkers or treatment targets for MNBAC and novel strategies for its treatment and prevention.

## Data Availability

The original contributions presented in the study are included in the article/Supplementary Material, further inquiries can be directed to the corresponding author.
